# Social support from teachers mediates physical activity behavior change in children participating in the Fit-4-Fun intervention

**DOI:** 10.1186/1479-5868-10-68

**Published:** 2013-05-28

**Authors:** Narelle Eather, Philip J Morgan, David R Lubans

**Affiliations:** 1 Priority Research Centre in Physical Activity and Nutrition, School of Education, Faculty of Education & Arts University of Newcastle, Callaghan Campus, Callaghan, Newcastle, NSW 2308, Australia

**Keywords:** Mediators, Physical activity, Children, School intervention, Health-related fitness

## Abstract

**Background:**

Few studies have examined the mediators of behavior change in successful school-based physical activity interventions. The aim of this study was to explore potential mediators of physical activity in the Fit-4-Fun program for primary school children.

**Design:**

Group randomized controlled trial.

**Methods:**

Four primary schools were recruited in April, 2011 and randomized by school into intervention or control conditions. Participants included 213 children (mean age = 10.7 years ± 0.6; 52.2% female) with the treatment group (n = 118) completing the 8-week multi-component Fit-4-Fun program. Participants were assessed at baseline, 3- and 6-months. Physical activity was measured using Yamax SW700 pedometers (mean steps/day) and questionnaires were used to assess constructs from Social Cognitive Theory and Competence Motivation Theory. Hypothesized mediators measured included social support from peers, parents and teachers; physical activity self-efficacy (barrier and task); enjoyment; and perceived school physical environment. Mediation was assessed using Preacher and Hayes’ multiple mediation regression SPSS macro. Action theory (A), conceptual theory (B) and the significance of the product of coefficients (AB) are reported.

**Results:**

The intervention had a significant effect on physical activity (p<0.001). The action theory test results revealed significant treatment effects at 3-months for perceived school environment (A=0.28, p<0.001); and at 6-month follow-up for perceived school environment (A=0.058, p<0.001), teacher social support (A=0.54, p<0.05) and enjoyment (A=-0.23, p<0.05). The conceptual theory test revealed a significant relationship between changes in teacher social support and changes in physical activity at 6-month follow-up (B=828, P<0.05). Teacher social support was shown to have a significant mediating effect on physical activity (AB = 445, CI = 77-1068 steps, proportion= 13%), and perceived school environment approached significance (AB = 434, CI= -415 to 1507 steps, proportion= 13%).

**Conclusions:**

The Fit-4-Fun program successfully targeted social support for physical activity provided by classroom teachers which contributed to improved physical activity in children. These results demonstrate that classroom teachers play a key role in influencing physical activity behavior outcomes in children.

Trial Registration No: ACTRN12611000976987

## Background

Physical activity is an important predictor of physical and psychological health in young people [1,2], and physical activity behaviors learned early in life may track through to adolescence and adulthood [3]. Yet research confirms that a large proportion of children do not participate in physical activity of sufficient volume and intensity to accrue the associated health benefits [4,5]. These trends highlight a need for implementing quality physical activity interventions that specifically facilitate the adoption of health-enhancing physical activity behaviors in children.

Schools have been universally identified as important institutions for the promotion of physical activity in children and youth [6], and quality health and physical education (HPE) is central to achieving physical activity goals in the school setting [7,8]. Consequently, a growing number of small [9]–[11] and large-scale [12] school-based physical activity interventions targeting young people have been implemented. Although these interventions have shown varied levels of success [9,12]–[15], there is limited understanding of the causal mechanisms of physical activity behavior change in school-based interventions [16].

As such, there is growing demand for researchers to explore and report mediators of physical activity change in youth interventions [16,17]. Mediation analysis can be used to expand our understanding of physical activity behavior change in children [18], as testing mediator variables allows researchers to determine which specific components of an intervention were linked to changes in physical activity behavior [19]. Building evidence around these determinants will guide future intervention development, implementation, evaluation and refinement.

A review of physical activity interventions that reported physical activity outcomes and potential mediators of behavioral change among children [17] identified 19 studies that reported both intervention effects on physical activity and mediators of behavior change (e.g., knowledge, self-efficacy, enjoyment, attitudes, behavioral capability, intentions, outcome expectancies, social norms, social support and self-concept) [17]. Although several of the reviewed trials reported intervention effects on mediators, none of the studies reported whether changes in these constructs mediated changes in children’s physical activity [17]. Similar conclusions were made by Lubans, Foster and Biddle (2008) and Demetriou and Honer (2012), in their reviews of school-based physical activity intervention studies in children and adolescents, with both reviews reporting a lack of quality mediation studies - making it hard to conclusively identify mediators of physical activity behavior change in children and in the school setting in particular [14,16]. More recently, Van Stralen and associates (2011) conducted a systematic review of mediating mechanisms in school-based energy behavior interventions [20], and found consistent evidence for self-efficacy as a mediator of treatment effects on physical activity behavior across 18 reviewed studies [20].

The application of behavioral theory is imperative when designing interventions for children as the theoretical constructs can help researchers determine how the intervention worked and how future interventions can be improved [21,22]. The Fit-4-Fun program was guided by the socio-ecological model and utilized the three critical components of the Health Promoting School (HPS) framework [23]. Socio-ecological models highlight the important role of the social and physical environment in determining behavior (and have demonstrated their potential for sustainable behavior change in youth interventions [24,25]), and the Health Promoting School (HPS) Framework is an important theoretical system for promoting health behaviors in the school-setting [23]. Social Cognitive Theory [26] and Competence Motivation Theory [27] are also two behavior theories that have been applied to the physical activity domain and assert that physical activity can be predicted by physical activity self-efficacy, support (social support and environmental support) and enjoyment [26,27]. By utilizing existing frameworks for facilitating behavior change and for creating supportive social and physical environments within the school and home, the Fit-4-Fun program aimed to address possible mediators of behavior change in relation to physical activity in children (e.g. social support, self-efficacy, supportive environment, enjoyment) [26,27].

The study protocols of the Fit-4-Fun program and the intervention effects on fitness and behavioral outcomes have been reported elsewhere [9,15]. The aim of the current study was to explore hypothesized mediators of physical activity behavior change in the Fit-4-Fun group randomized controlled trial [28].

## Methods

### Study design and participants

The Fit-4-Fun program was evaluated using a group RCT with 226 Stage 3 (Grade 5 and 6) students from four primary schools (mean age 10.7 ± 0.6 years; 52.2% female) located in the Hunter Region, NSW, Australia. There were 10 teachers from the 4 schools (2 × treatment and 2 × control schools), with 5 classes in each study group (3 × year 6 and 3 × year 5 in each). Schools were randomized into the Fit-4-Fun treatment (n=118) or wait-list control conditions (n=108) following baseline assessments. The random allocation sequence was generated by a computer-based random number-producing algorithm and completed by a researcher not involved in the project to ensure an equal chance of allocation to each group. Assessments were conducted in April (baseline), June (3-month follow up) and December (6-month follow-up), 2011, and completed by trained research assistants who were blinded to treatment conditions at baseline assessments. The study was registered with the Australia and New Zealand Clinical Trials registry (ACTRN12611000976987). Ethics approval for this study was obtained from the University of Newcastle, NSW, Australia and the Newcastle-Maitland Catholic Schools Office, and school Principals, teachers, parents and study participants provided written informed consent. The methods of the Fit-4-Fun study have been reported in detail elsewhere [28], with the trial being adequately powered to detect group changes in the primary outcome cardio-respiratory fitness (CRF)(VO2max) based on a previous study by Kolle et.al (2009). In addition, the study was adequately powered to detect a between group difference of 1500 steps and medium-sized mediation effects using a product-of-coefficients test [29].

### Treatment conditions

The Fit-4-Fun intervention was informed by the Fit-4-Fun pilot study [9] and a detailed description of the intervention has been reported previously [28]. All of the control schools and treatment schools had the same time allocation for physical education (60min per week) and for recess and lunch breaks (20min recess and 40 min lunch).

a) Fit-4-Fun Intervention

*Theoretical framework*: The Fit-4-Fun Program was grounded in Bandura’s Social Cognitive Theory (SCT) and Harter’s Competence Motivation Theory (CMT) and aimed to provide children with the knowledge and skills necessary for short- and long-term PA behavior change [27]. The program aimed to promote the development and maintenance of positive physical activity behaviors and attitudes among participants, by targeting possible mediators of behavior change (including social support, self-efficacy, supportive environment, enjoyment) [27,30].

Firstly, a selection of engaging physical activities, games, challenges and learning activities were included in the program to improve “enjoyment” of physical activity, as the level of enjoyment experienced during physical activity is considered one of the most important reasons that children become involved and to continue to participate in physical activity - and a lack of fun or enjoyment is likely to lead them to withdraw [17,31]. Secondly, techniques shown to positively influence physical activity self-efficacy were embedded in the Fit-4-Fun program, as self-efficacy beliefs have been shown to directly and indirectly influence motivation, affect and behavior [30], and associates with daily vigorous physical activity levels in children and youth [32]–[34]. Thirdly, previously tested strategies to improve physical activity levels by improving the schools’ physical environment were employed [35]–[37]. In addition, social support for participation in the program activities provided by classroom teachers, parents, and students was a targeted strategy in the Fit-4-Fun program, as social support has been positively associated with physical activity participation in youth [38]–[41]. Support for participation in physical activity, in the form of encouragement, was provided verbally by parents, classroom teachers and peers (e.g. teachers prompted children to join in the break-time games as they exited the classroom). Visual aids such as posters pinned on the classroom doors, school newsletter articles, the student work booklets, and a reward system were also utilized to provide support for the program and encourage participants to engage in the program. A full description of the intervention components, the behavior change techniques and targeted constructs are provided in Table 1. A unique feature of the Fit-4-Fun Program was that it encouraged children to participate in vigorous intensity physical activity using games, challenges and learning experiences that were “fun” or enjoyable and that appealed to children [28].

**Table 1:** ‘Fit-4-Fun’ program content and alignment with theoretical constructs (Australia, 2011)

**Wk**	**Session focus**	**Session overview**	**Behavior change strategies**	**SCT** / **CMT constructs**
**1**	Health-related fitness (theory)	• Program rational	• Provide information about PA & PF behaviors / link to health	• Outcome expectations
		• Defining PA & PF		
		• HRF & SRF	• Develop self-monitoring skills (weekly PA timetable, talk test)	
		• PA guidelines		• Social support (home & school)
		• Analyzing current PA & PF behaviors	• Provide social support and encouragement (to meet PA guidelines)	
			• Participate in age-specific “fun” physical fitness activities (HW task)	• Self-efficacy
			• Develop goal setting skills (HW task)	• Intentions
			• Provide equipment and task cards for use during recess and lunch breaks	• Motivation
				• Enjoyment
				• School environment
**2**	Cardio-respiratory fitness (CRF) (theory & practical)	• Provide information on CRF	• Provide information about CRF & the role of the heart & lungs during PA	• Outcome expectations
		• Role of heart & lungs during PA	• Participate in physical fitness practical laboratory	• Self-efficacy
		• Linking heart rate (HR) to PA intensity (lab)	• Develop skills in self-monitoring (using heart rate)	• Social support
		• Linking CRF & health	• Predicting consequences of actions	• Motivation
			• Making recommendations relating to PA and CF	• Enjoyment
			• Participate in age-specific “fun” physical fitness activities (HW task)	• School environment
			• Provide equipment and task cards for use during recess and lunch breaks	
**3**	Improving cardio-respiratory fitness (practical)	• Revise CRF & measuring intensity using HR	• Provide opportunity to participate in enjoyable physical activities in a supportive environment	• Outcome expectations
		• Participate in a practical PE lesson with a gross motor warm-up activity, dynamic stretches, skill development activities, modified games and cool-down	• Maximal participation is provided for and encouraged	• Social support
			• Positive feedback is provided throughout the session	• Self efficacy
			• Students are to reflect on their performance and re-assess current PA behaviors	• Motivation
		• HR is monitored throughout the lesson	• Participate in age-specific “fun” physical fitness activities (HW task)	• Enjoyment
		• Discussion about the type of PA and heart rate (high intensity / vigorous)	• Provide equipment and task cards for use during recess and lunch breaks	• School environment
			• Provide information on MF	
**4**	Muscular Fitness (MF) (theory & practical)	• Define MF	• Link current PA behavior to MF	• Outcome expectations
		• Muscular strength Vs Muscular endurance	• Develop goal setting skills / set targets to achieve	• Social support
		• Activities that require MF	• Self-monitoring skills (PF tests)	• Self-efficacy
		• Measuring MF (lab)	• Participation in non-threatening practical assessments	• Intentions
				• Motivation
				• Enjoyment
		• Linking MF & health Improving MF	• Participate in age-specific “fun” physical fitness activities (HW task)	• School environment
			• Provide equipment and task cards for use during recess and lunch breaks	
**5**	Improving muscular fitness (practical)	• Revise MF & measuring MF	• Provide opportunity to participate in enjoyable physical activities in a supportive environment	• Outcome expectations
		• Participate in a practical PE lesson with a gross motor warm-up activity, dynamic stretches, MF circuit and cool-down	• Maximal participation is provided for and encouraged	• Social support
			• Positive feedback is provided throughout the session	• Self-efficacy
		• HR is monitored throughout the lesson	• Students are to reflect on their performance and re-assess current PA behaviors	• Motivation
		• Discussion about the type of PA and MF (e.g. resistance training)	• Participate in age-specific “fun” physical fitness activities (HW task)	• Enjoyment
			• Develop goal setting skills (HW task)	• School environment
			• Provide equipment and task cards for use during recess and lunch breaks	
**6**	Flexibility (theory & practical)	• Define flexibility	• Provide information on flexibility	• Outcome expectations
		• Activities that require MF	• Link current PA behavior to flexibility	
		• Benefits of being flexible	• Develop goal setting skills / set targets to achieve	• Social support
		• Types of stretching	• Self-monitoring skills (PF tests)	• Self-efficacy
		• Improving flexibility (lab)	• Participation in non-threatening practical assessments	• Intentions
		• Linking MF & health	• Participate in age-specific “fun” physical fitness activities (HW task)	• Motivation
		• Improving MF	• Provide equipment and task cards for use during recess and lunch breaks	• Enjoyment
		• Predicting outcomes from changed MF behaviors		• School environment
		• Goal setting task		
		• Link flexibility to lifestyle behaviors		
**7**	Improving flexibility (practical)	• Revise flexibility and measuring flexibility	• Provide opportunity to participate in enjoyable physical activities in a supportive environment	• Outcome expectations
		• Participate in a practical PE lesson with a gross motor warm-up activity, dynamic stretches, fun stretching routines and cool-down	• Maximal participation is provided for and encouraged	• Social support
			• Positive feedback is provided throughout the session	• Self-efficacy
		• HR is monitored throughout the lesson	• Students are to reflect on their performance and re-assess current PA behaviors	• Motivation
		• Discussion about the type of PA and improved flexibility	• Link to lifelong behaviors	• Enjoyment
			• Participate in age-specific “fun” physical fitness activities (HW task)	• School environment
			• Provide equipment and task cards for use during recess and lunch breaks	
**8**	Improving health-related fitness through games (practical)	• Revise HRF components	• Provide opportunity to participate in enjoyable physical activities in a supportive environment	• Outcome expectations
		• Revise improving HRF		
		• Participate in a student-centered practical PE lesson where students adapt fun games to incorporate HRF	• Maximal participation is provided for and encouraged	• Self-efficacy
		• HR is monitored throughout the lesson	• Positive feedback is provided throughout the session	• Social Support
		• Discussion about the type of PA and improved HRF	• Students learn skills in adapting PA to improve HRF	• Motivation
		• Summary of health benefits with improved HRF	• Students are to reflect on their performance and re-assess current PA behaviors	• Enjoyment
		• Evaluation of ‘Fit-4-Fun’	• Link to lifelong behaviors	• School environment
			• Participate in age-specific “fun” physical fitness activities (HW task)	
			• Reflection Task (HW task)	
			• Provide equipment and task cards for use during recess and lunch breaks	
**1**-**8**	‘Fit-4-Fun’ Home Activities	• Participation in an 8 week home activity program	• Students participate in a range of fun activities with their parents / siblings	• Outcome expectations
		• 2 weekdays: MF, flexibility, CRF activities	• Family provide social support throughout the program	• Self-efficacy
			• Students develop skills in self-monitoring and self-motivating	• Social Support
		• 1 weekday: fitness assessments	• Students develop skills in goal setting & time management	• Motivation Enjoyment
		• Weekends: family activities & CRF assessment	• Students develop skills in assessing & planning to improve the physical environment (assessment task)	
		• Weeks 1, 5, 8: Goal setting tasks		
		• Problem Solving Task (assessment)		
**1**-**8**	Daily break time (recess and lunch) activities	• Student-directed activities and tasks for use during school break times (e.g. small sided games, challenges and strength activities using playground equipment)	• Provide opportunity to participate in enjoyable physical activities in a supportive environment	• Self-efficacy
				• Social Support
			• Maximal participation is provided for and encouraged by peers	• Enjoyment
		• Laminated Task Cards and equipment supplied	• Students learn skills in self-motivation / regulation Link to lifelong behaviors	• School environment
		• Participation will be assessed via self-report at 3-month follow-up		

Abbreviations: *SCT* Social Cognitive Theory, *CMT* Competence Motivation Theory, *HRF* Health-Related Fitness, *HR* Heart rate, *CRF* Cardio-respiratory fitness, *MF* Muscular fitness, *PA* Physical activity, *PF* physical fitness, *HW* homework.

The Fit-4-Fun Program included three major components based on the HPS Framework [23]:

*Curriculum program*: An 8-week × 60-min HPE program based on the NSW K-6 syllabus [42] was delivered during normal HPE lesson time [42]. The program was designed to improve understanding and a range of skills in relation to physical activity and fitness (including skills in assessing and monitoring physical activity and HRF levels). The program overview has been summarized in Table 1. The Fit-4-Fun program was delivered by a member of the research team who is an experienced physical educator.

*Family partnership*: Children, their parents and family members were provided with an 8-week home activity program designed to improve the duration, type and intensity of physical activity performed at home using a range of engaging and enjoyable fitness activities, small-sided games and fitness challenges (3 × 20 min per week for 8 weeks). Children were given a range of physical activities to choose from, and were encouraged to select activities from each of the physical activity categories (muscular fitness, flexibility and cardio-respiratory fitness). There were also goal setting activities and reflection tasks for students to complete with their parents throughout the program, enabling them to set personal fitness goals, monitor their achievement and to reflect on their progress.

*School environment*: Students were encouraged to participate in physical activity during recess and lunch each day. To encourage students to be active during this time, schools were provided with activity task cards outlining the rules and organization of a range of fun and vigorous games (e.g. small-sided invasion games, skipping challenges) and a variety of equipment ( e.g. balls, markers, skipping ropes) for use during break-times. This initiative was student-directed and students were asked to support their friends throughout the program by encouraging them to join in the activities and by working together to organize games.

*b*) *Control* (*wait*- *list control group*): The control group participated in their usual 60 min / week HPE lesson over the 8-week intervention period delivered by their normal classroom teacher. The lesson content was determined by the existing school HPE program. The control group received the Fit-4-Fun program resources after the 6-month assessment.

### Measures

Trained research assistants conducted all assessments, which were completed at the study schools using the same instruments at each time point.

*Physical activity*: Participants wore a sealed Yamax SW700 pedometer (Yamax Corporation, Kumamoto City, Japan) for 7 days (including at least three consecutive days and one weekend day) [43] to determine their physical activity levels. Yamax pedometers have been shown to be a valid and reliable objective measure of physical activity in children [44,45]. Pedometer placement was standardized by placing it on the belt or waistband, in the midline of the thigh. Participants were instructed to put the pedometers on each morning and to leave it on until they went to bed (except when showering or performing water-based activities). To minimize the amount of lost data (i) teachers recorded participants’ results on a recording sheet and then reset the pedometer at the same time each morning, (ii) on weekends an information and recording sheet was sent home to parents to complete each morning, and (iii) teachers were asked to frequently remind students to wear their pedometer during all waking hours. Non-wearing periods (e.g. during participation in water sports), were recorded and adjusted for via imputation (1000 steps for 10 minutes of moderate –vigorous activity and 1500 steps for vigorous activity) [44].

### Student questionnaire

Participants completed a questionnaire at baseline, 3-month follow-up and 6-month follow-up, which was designed to collect information about the attitudes, opinions, behaviors and characteristics of the children. The questionnaire design and purpose is described below.

I. *Demographic information*: age, sex, language spoken at home and country of birth. 

II. *Fitness testing experience*: Information relating to participants’ experience with fitness testing was sought through the use of five structured closed and semi-closed questions (e.g. *Have you ever participated in a fitness test*?)

III. *Theoretical constructs*: Table 2 provides a description of the hypothesized mediator scales (i.e., self-efficacy, enjoyments, social support and physical activity environment), the psychometric properties of each scale, and the previously reported reliability and validity data. The mean score for each participant on each scale was calculated at each of the three assessment time-points.

**Table 2:** **Description and psychometric properties of hypothesized mediator scales** (**Australia**, **2011**)

	**Description of scale**	**Range (no. of items)**	**Source**	**α**
**Barrier self-efficacy**	* Single factor 5-point Likert format	1-5	Adapted version of an 8-item questionnaire previously developed for use with 5th, 8th and 9th grade girls (PASES) [46]–[48]. The modified scale has been shown to be a valid measure of barrier self-efficacy for this age group (α=.81, ICC=.57) [49], and confirmatory factor analysis showed good fit for use with 6^th^ and 8^th^ grade girls (CFI=.98; CFI=0.99) [47]. Factor structure, loadings, factor variance, item means and errors were shown to be invariant across age groups and race / ethnic groups (SE=0.4, 0.024, p<0.001) [50,51], with good test-retest stability (.84) [49].	1= .75
	* Participants were asked to select how much they agree with the eight statements by ticking the relevant circle	8 items		
	* E.g. *I can be physically active even if it is hot or cold outside*).			
	* Scale: 1=Disagree a lot to 5= Agree a lot			
**Enjoyment**	* 5-point Likert format The child was asked to select how often they experience the relevant feeling about physical activity by ticking the relevant circle	1-5	Adapted version of the a 16-item version of the Physical Activity Enjoyment Scale (PACES) [52] and has been recently validated for use with children (CFI=0.95), with good stability across age groups (SE=0.03;0.24, p<0.001) [52]–[54] and good test-retest stability (.73) [49].	1= .72
		6 items		
	* E.g. *When I am physically active*…….…. *It*’*s no fun at all*	(negatively worded)		
	* Scale: 1=Never to 5= Every day			
**Social Support family peers/friends teacher**	* 5-point Likert format	1-5	Adapted scale based on two scales used in the student survey of the Amherst Health and Activity Study [55]. Recently tested for validity and use with children in the 6^th^ and 8^th^ grade by Dishman and colleagues (family and friend scales only) [51]. Validity measures indicate that the factor structure, factor loadings and factor variances / co-variances were invariant across race/ethnic groups and across age groups and across time (CFI=0.96; 0.98, SE (friends) =0.41; .027 and SE (family) =0.53; 0.021, p<0.001). The teacher social support scale was devised for the purpose of this study and follows the structure and wording of the family and friends social support for physical activity scales [51].	P1= .68
	* Participants were asked to select how often a specific form of social support (encouragement or modeling) is provided to them during a typical week by ticking the relevant circle	3 scales		
				F1= .65
		Peers (P)		
		(3 items)		
		Family (F)		T1= .77
		(4 items)		
		Teacher (T)		
		(4 items)		
	* E.g. Modeling: *During a typical week at school*, *how often do your FRIENDS*.... *do physical activity or play sports with you*?			
	* E.g. Encouragement: *During a typical week at school*, *how often does your TEACHER*.... *encourage you to do physical activity during recess or lunch breaks*?			
	* Scale: Never = 1 to Every day = 5			
**Perceived school enviornment**	* Single factor 4-point Likert format	1-4	Adapted version of the 2-factor, 20-item questionnaire Q-SPACE developed by Robertson-Wilson, Levesque and Holden [56]. Initial findings support the reliability (α=0.86 and test-retest reliability=0.78) and construct validity of the physical environment sub-scale for use with children and youth [56,57].	1= .80
	* The participant was asked to select how much they agree with the eight statements by ticking the relevant circle	9 items		
	* E.g. *There is sports equipment available for students to use during recess and lunch breaks*			
	* Scale: 1= Strongly Disagree to 4= Strongly Agree (no neutral response)			

### Statistical analysis

**Figure 1:**
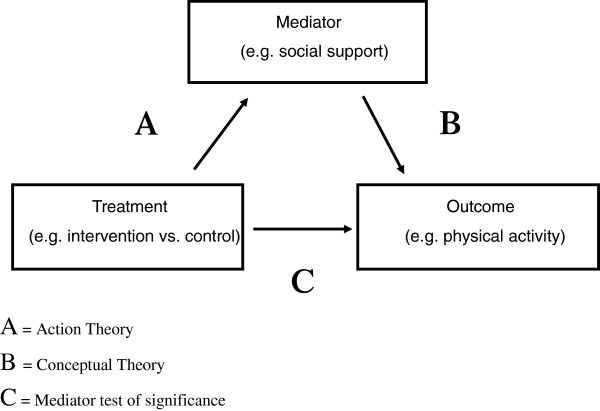
Mediation analysis overview.

A range of statistical methods are commonly used in mediation analyses (e.g. Baron and Kenny -causal steps approach, Alwin & Hauser - Product-of-coefficients method, and Judd and Kenny - difference in-coefficients) [58,59]. These methods generally consist of an Action Theory test, a Conceptual Theory test and a Significance Test of the mediated effect (MacKinnon, 2008). In summary, the Action theory test examines the impact of the intervention on the hypothesized mediators (e.g. social support, enjoyment, physical activity self-efficacy), the conceptual theory test investigates the relationship between changes in hypothesized mediators and changes in the targeted behavior (e.g. physical activity), and the significance test combines the action and conceptual theory tests to determine the significance of the mediated effect (see Figure 1 below) [58].

Preacher and Hayes’ Multiple Mediation Regression [60] macro for IBM SPSS (version 19.0) was used to perform the mediation analyses. This method was determined to be most appropriate given that four level 2 units (schools) were insufficient to provide a reliable estimate of between-school variance using multi-level regression [61,62]. The Preacher and Hayes macro performs all of the mediation steps simultaneously, but to highlight the output that it generates, the steps are outlined below. Single and multiple (i.e., models included all potential mediators) mediator models were tested and all analyses were adjusted for baseline values. In step 1, the total effect of the intervention on physical activity was estimated by regressing physical activity onto treatment condition (intervention or control; C coefficient). Step 2 was the Action Theory test, which involves regressing the potential mediators onto treatment condition (A coefficient). Step 3 was the Conceptual Theory test, which involved regressing physical activity onto treatment condition (C´ coefficient) and mediators (B coefficient). Step 4, the significance of the product-of-coefficients (AB) was tested by computing the associated asymmetric bias-corrected bootstrap confidence intervals using the INDIRECT add-on for SPSS [60]. Finally, asymmetric confidence intervals were to determine the significance of the product-of-coefficients (AB) For a variable to satisfy the criteria for mediation the 95% confidence intervals (CI) for the product-of-coefficients (AB) must not include zero. The proportion of the total effect that was mediated was also calculated [AB/ (C´ +AB)]. The assessment of mediation immediate post-intervention was performed to determine if there were changes in theoretical constructs – and whether they mediated any change in physical activity levels. The assessment of mediation at 6month follow-up was also performed to determine whether possible mediators where present (and whether they had changed) even though the program had finished and the research team no longer had contact with the school.

## Results

Overview: Participants included 213 children (mean age = 10.7 years ± 0.6; 52.2% female) with the treatment group (n=118) completing the 8-week Fit-4-Fun Program. Participants were assessed at baseline and 6-month follow-up, with a 91% retention rate (9% were absent on the day of 6-month assessments). Of the 213 participants, 93.5% were born in Australia and 98.1% spoke English at home. At baseline, there were no significant differences between groups for demographic variables (gender, age, country of birth, primary language). A detailed description of the participants’ demographics has been reported previously [15].

The intervention effects for health-related fitness and physical activity have been reported previously [15]. In summary, after 6-months, significant treatment effects were evident in cardio-respiratory fitness (adjusted mean difference = 1.14 levels, p<0.001), body composition (BMI, -0.96 kg/m^2^, p<0.001 and BMI z-score, 0.47 z-scores, p<0.001), flexibility (sit & reach mean, 1.52cm, p=0.0013), muscular fitness (7-stage sit-up, 0.6 stages, p=0.003) and physical activity (3253 steps/day, p<0.001). There were no group by time effects for three measures of muscular fitness (basketball throw, push-ups and standing jump).

### 3-month results

I. Action theory test: significant improvement in the treatment group for perception of the school physical environment (A=0.28, p<0.001) was evident at 3-months (Table 3). There were no significant difference in scores for physical activity self-efficacy, enjoyment, social support from family, friends and teachers (p>0.05).

II. Conceptual theory: there were no significant relationships between changes in the hypothesized mediators and changes in physical activity levels at 3-months (p>0.05).

III. Significance test of mediated effect: none of the hypothesized mediators met the criteria for mediation at 3-months.

**Table 3:** **Action theory test**, **conceptual theory test and significance of the mediated effect on physical activity** (**step count**) – **Baseline to 3**-**months** (**April** - **June**, **2011**) **and baseline to 6**-**months** (**April** - **December**, **2011**) **Australia**

**Hypothesized mediators**	**Time**	**Action theory test^**		**Conceptual theory test**		**Direct effect**		**Indirect effect**	**95% CI**	**Proportion (%)**
		**A (SE)**	**p-value**	**B (SE)**	**p**-**value**	**C’ (SE)**	**p-value**	**AB (SE)**		**AB/(C’ + AB)**
**Self Efficacy**	1	0.04 (0.08)	0.62	1012 (635)	0.11	4368 (621)	<0.001	41 (107)	−107 to 357	1%
	2	0.18 (0.11)	0.10	1081 (587)	0.68	3168 (725)	<0.001	190 (168)	−9 to 717	6%
**Enjoyment**	1	−0.16 (0.11)	0.14	860 (487)	0.08	4233 (625)	<0.001	−140 (138)	−697 to 19	3%
	2	−0.23 (0.11)	0.05	1002 (527)	0.06	3775 (688)	<0.001	−226 (168)	−732 to 7	6%
**Social Support** (**Friends**)	1	0.01 (0.12)	0.91	407 (446)	0.36	4034 (605)	<0.001	−90 (70)	−89 to 247	2%
	2	0.21 (0.14)	0.14	497 (426)	0.25	3423 (703)	<0.001	106 (125)	−34 to 573	3%
**Social Support** (**Family**)	1	0.03 (0.11)	0.82	612 (450)	0.18	3911 (605)	<0.001	16 (86)	−112 to 266	<1%
	2	0.06 (0.12)	0.64	516 (515)	0.32	3402 (694)	<0.001	29 (102)	−100 to 411	<1%
**Social Support** (**Teacher**)	1	−0.12 (0.15)	0.43	−257 (337)	0.45	3937 (617)	<0.001	32 (78)	−49 to 357	<1%
	2	0.54 (0.17)	<0.001	828 (369)	0.03	3037 (714)	<0.001	445 (242)	77 to 1068	13%
**School Environment**	1	0.28 (0.07)	<0.001	−605 (733)	0.41	4037 (680)	<0.001	−172 (187)	−574 to 173	4%
	2	0.58 (0.09)	<0.001	742 (723)	0.31	2933 (836)	<0.001	434 (459)	−415 to 1507	13%
**Multi**-**mediation** (**all**)	2					3009 (894)	<0.001	390 (570)	−658 to 1714	11%

1= baseline to 3-month.

2= baseline to 6-month.

*Note*. Control and intervention groups were coded ‘0’ and ‘1’ respectively; A = estimate of unstandardized regression coefficient of treatment condition predicting change in hypothesized mediators; B = estimate of unstandardized regression coefficient of change in hypothesized mediators predicting change in physical activity behavior, *AB* = product of coefficients estimate, *C*’ = effect of treatment condition on physical activity behavior controlling for mediator effect, *SE* = standard error, 95% CI = asymmetric bias-corrected bootstrap 95% confidence interval.

### 6-month results

I. Action theory test: significant changes in perceived school environment (A=0.58, p<0.001), teacher social support (A=0.54, p<0.05) and enjoyment (A=-0.23, p<0.05) were evident at 6-month follow-up (Table 3). There were no significant difference in scores for self-efficacy, and social support from family or friends (p>0.05).

II. Conceptual theory: a significant relationship between changes in teacher social support and changes in physical activity levels at 6-month follow-up (B=828, P<0.05) were recorded. There were no significant relationships between changes in the hypothesized mediators (p>0.05) at 6-months.

III. Significance test of mediated effect: at 6-month follow-up teacher social support was shown to have a significant mediating effect on physical activity (C=445, CI=77-1068, proportion=13%). Perceived school environment approached significance (C=4037, CI=-415 to 1507, proportion= 13%), while social support form peers and parents, self-efficacy and enjoyment did not meet the conditions for mediation (p>0.05).

Multiple mediator models

I. Action theory test: significant changes in perceived school environment (A=0.28 p<0.001) and teacher social support (A=0.47, p<0.05) were evident at 6-month follow-up. There were no significant difference in scores for self-efficacy, social support from family or friends (p>0.05) or enjoyment.

II. Conceptual theory: a significant relationship between changes in teacher social support and changes in physical activity levels at 6-month follow-up (B=863, P<0.05) were recorded. There were no significant relationships between changes in the hypothesized mediators (p>0.05) at 6-months.

III. Significance test of mediated effect: None of the constructs satisfied the criteria for mediation at 6-month follow-up in the multiple mediator models.

## Discussion

The primary objective of this study was to identify if constructs from SCT and CMT mediated changes in physical activity in the Fit-4-Fun school-based intervention. This study demonstrated that the social support provided by classroom teachers mediated the effect of the Fit-4-Fun intervention on physical activity. No other constructs satisfied the criteria for mediation.

Social support is considered an important determinant of behavior in socio-ecological models and behavioral theories such as SCT [63] and CMT [27]. Importantly, the Fit-4-Fun program included a range of strategies to increase the amount of social support for physical activity provided by the classroom teachers. The findings in this study support the pivotal role teachers have in the promotion of physical activity in schools, on learning in physical education and influencing physical activity levels in children. This is consistent with previous work which has highlighted the relationship between the schools’ social environments and children’s physical activity behaviors [56,64]–[68]. Teachers in the intervention schools did not allocate additional time for physical activity during the school day, but were encouraged to provide regular support for participation in physical activity (via verbal encouragement during physical education lessons and daily classroom activities, and via school wide promotion strategies such as newsletters, assemblies, and posters displayed around the school). Furthermore, changes in the teachers modeling of positive physical activity behavior may have influence perceptions of support by participants, and hence physical activity levels, as the teachers involved would have also developed professionally due to their involvement in the program. Studies have also shown that teachers are able to enhance students’ intrinsic motivation for physical activity and their perceived athletic competence when they support physical activity goals and provide positive feedback in a stimulating and supportive classroom environment [69,70]. Furthermore, teachers have an influential role on learning in the general classroom environment, where the teacher’s knowledge, behaviors, and opinions have been shown to be very powerful in the learning equation (accounting for approximately 30% of the variance in learning) [71]. Consequently, it could be anticipated that this influence would also project into learning outcomes in physical education.

In this study, a mediation affect was found at 6-month follow-up and not at immediate post-intervention, possibly indicating that the general classroom teacher had increased their confidence and skills to promote high levels of physical activity with their students, and consequently took on the responsibility for providing support for physical activity once the specialist had finished delivering the face-to-face curriculum program. During the Fit-4-fun program, the classroom teachers were not responsible for delivering any aspect of the Fit-4Fun program but were able to observe lessons and assist in some minor aspects of set up and student management. Having a highly experienced and qualified physical education teacher come into the school to take the students for an hour each week for PE would have given the teachers the opportunity to observe quality physical education classes and thus provide a unique opportunity for professional development. On the contrary, once the PE specialist had finished the program, the classroom teacher resumed all responsibility for the design, delivery and support for physical activity programs within the school. It is thus plausible that the children recognized and valued the more influential role of their classroom teachers in the period following the program and this support was clearly instrumental in providing the necessary motivation and opportunities for a sustained intervention impact.

Social support from parents did not mediate physical activity behaviors in this study. Our results align with previous school-based physical activity interventions, which have found little evidence for the mediating effect of parents’ social support for physical activity on their children’s activity levels [68,72]–[74]. The literature consistently refers to the important role that parents and families have on health behaviors, especially physical activity [40,75], and that researchers have had difficulty in engaging parents in physical activity programs in the past [76]. Consequently, specific measures were taken to engage parents and family members in the Fit-4-Fun program. Parents in the Fit-4-Fun study were given written information about the study via notes, newsletters and information booklets, were invited to attend a parent-child fitness session after school, and were encouraged to participate in the 8-week homework activity program with their child. Kipping (2011) showed that homework tasks are a feasible way of involving parents and that they can serve a range of purposes [77]. In this intervention, homework was designed to encourage children and their parents to participate in a range of enjoyable physical activities together, to learn how to monitor and improve their fitness levels and to encourage families to support each other in achieving physical activity goals. As previously reported [15], parental support for the program was minimal and many children reported lack of involvement by parents. Research has identified the challenges with using homework as a method of involving parents, with many parents lacking time, knowledge, guidance and motivation to support children out of school hours [78]. However, given that parents take on the responsibility of being role models, sources of encouragement, and facilitators of physical activity for children [79,80], it is important to continue to investigate methods of using engaging homework tasks or other strategies to promote physical activity behaviors among children [81].

Social support for physical activity from friends did not exhibit mediating effects on physical activity behaviors in this study. Research in this area is sparse, with many investigators evaluating the important role of peers as a moderator of social and emotional development [82], rather than physical activity behaviors. One investigation by Salvy (2008) examined the associations between children’s physical activity and peers, and found that the presence of peers and friends is associated with higher activity intensity [83]. A more recent study by Lubans, Morgan and Callister explored the potential mediation effects of peer support on physical activity behaviors in adolescent boys and reported that peer support did not meet the criteria for mediation in their study [84]. The PALs program implemented a ‘Student Leader’ system, whereby students took on the role of organizing physical activities sessions for their peers and for younger students, and of providing support and encouragement for participation in these physical activities sessions [84]. A possible explanation for the PALs’ findings could be linked to participants’ ‘heightened awareness’, whereby students become more aware of the support they are not receiving – affecting follow-up data. The potential to utilize ‘Student Leaders’ to improve physical activity levels in the younger age group was explored in the Fit-4-Fun study, with students given the opportunity to take on the role of ‘Student Leader’ during break times. The role entailed encouraging classmates to be active at recess and lunch, collecting the equipment for use during break times, and taking the break-time game cards out into the playground each day (for a period of 2 weeks). In general, the children in the Fit-4-Fun study did not embrace this system and this aspect of the intervention was poorly implemented. Further investigation into designing appropriate strategies to engage young people and teachers in promoting activities during break-time and to increase social support from peers should be considered.

Contrary to recent data suggesting that both self-efficacy and enjoyment are positively associated with physical activity in children and youth [16,17,20,32]–[34,51,85,86], neither variable satisfied the criteria for mediation in the current study. Self-efficacy is the most commonly assessed mediator and receives the strongest support for mediating the relationship between school-based interventions and physical activity in young people [14,16,17,20]. In this study, it was envisaged that targeting self efficacy would directly and indirectly influence motivation, affect and physical activity, respectively [30,87]. Although several strategies were used in the Fit-4-Fun program to develop physical activity self-efficacy (e.g. goal setting, positive reinforcement for effort or progress towards a set behavior, the provision of instruction and feedback on performance, self-monitoring, self- regulation, the provision of information on consequences of behavior, and skills practice) and enjoyment (e.g. the inclusion of “fun” and engaging physical activities, games, challenges and learning activities), a ceiling effect may have nullified our analyses. The relatively high self-efficacy scores (mean self-efficacy baseline = 4.23 / 5.00) and enjoyment scores (mean enjoyment baseline 4.41 / 5.00) at baseline, implies that the children had relatively high levels of confidence in their ability to perform physical activities and enjoyed participating – limiting the scope of the intervention to improve these constructs. Recent data also suggests that enjoyment is positively associated with physical activity in children and youth, yet we did not exhibit an intervention effect for enjoyment or satisfy the criteria for mediation in this study. Multiple strategies were implemented in the Fit-4-Fun program to improve enjoyment of physical activity (e.g. the inclusion of “fun” and engaging physical activities, games, challenges and learning activities), but the high baseline enjoyment scores (mean enjoyment baseline 4.41 / 5.00) indicate that the children already enjoyed participating in physical activity– creating a likely ceiling effect and limiting the scope of the intervention to improve this targeted construct. Alternatively, the limited/negative impact on enjoyment may be explained by the intense nature of the physical activities utilized in the Fit-4-Fun program. Research by Schneider and associates [88,89], has shown that the proportion of young people who experience a positive enjoyment affective response to hard exercise is relatively small and although the activities in the Fit-4-Fun program were specifically designed to maximize enjoyment, they still required participants to work vigorously and to perform ‘hard’ muscular fitness activities – potentially perceived as less enjoyable than “easier” or less intense physical activities. Furthermore, the enjoyment scale used in the student questionnaire (adapted version of the 16-item Physical Activity Enjoyment Scale (PACES) [54] may not have been suitable for capturing true intervention effects in this study. The 6 questions in the enjoyment scale did not target specific types of physical activity or differentiate between physical activity settings – making it difficult to establish whether these changes in enjoyment are a result of ‘response shift’ (where a child’s perception of enjoyable physical activity changes as a result of experiencing new and more enjoyable activities). However, given that the mean scores for enjoyment were found to be greater than 4 at all three assessment time points (indicating that most children answered “never” or “once” to negative feelings during physical activity), this could be viewed as a positive result. The design and validation of specific scales assessing enjoyment of physical activity in specific settings and at different time periods throughout the week are clearly needed.

Furthermore, children are generally optimistic about their abilities, but these start to decline during adolescence. This was demonstrated in another physical activity intervention by Lubans et al. (2010), who also found that physical activity self-efficacy did not mediate changes in physical activity behavior in adolescents [73]. However, it is worth noting that the distinction between barrier self-efficacy (confidence to overcome a barrier) and task self-efficacy (confidence to perform a task) is often overlooked, with most physical activity interventions assessing barrier self-efficacy only (which children might have difficulty recognizing) [87]. In the current study, a general self-efficacy scale was used, where barrier and task self-efficacy were assessed simultaneously. In the PALs study [84], there was a significant impact on task self-efficacy, implying that perhaps more physical activity studies involving young people (especially children) should explore students’ confidence in their skills. In addition, the use of existing self-efficacy scales (whether they focus on barrier self-efficacy, task self-efficacy or both) may not be capturing the true effect of physical activity interventions – especially in children. The design and use of specific scales assessing both self-efficacy constructs (independently) may provide a better insight into the factors mediating physical activity behaviors in children.

The school’s physical environment is also an area that has received much attention in promoting physical activity in the school setting [24,74]. The provision of adequate space, playground equipment, non-fixed sports equipment and non-curricular opportunities during break times in the school day (recess and lunch), has shown to relate to the amount and intensity of physical activity that school children participate in during these times [90]–[93]. Our intervention results indicated that participants’ ratings of their school physical environments declined from baseline to 3-month, and from baseline to 6-month follow-up. Possible explanations for these results may relate to the suitability of the school environment scale used in the student questionnaire (and it ability to reflect the intervention components designed to change physical activity behavior), to the ceiling effect created by the relatively high participant scores at baseline, [94]. In the Fit-4-Fun study, it is possible that participants in the intervention group became increasingly aware of how to increase their physical activity levels during break times and sought opportunities to do so. However, although schools were encouraged to provide access to sports equipment during breaks and additional sports equipment was provided to schools (e.g. balls, skipping ropes), limited changes to the fixed play equipment (both intervention schools had considerable existing climbing equipment, undercover play areas, playground markings and target equipment) and to the size of the playground, would have hampered the potential to improve ratings in these areas.

### Study strengths and limitations

This study has noteworthy strengths that include: a novel intervention targeting physical activity and fitness in children, a high quality trial that adhered to the CONSORT statement [95], excellent study adherence and participant retention and the assessment of physical activity using an objective measure. However, there are some study limitations that should be observed. Firstly, due to the process of randomization by school and the small number of clusters (four schools), statistical analysis were not adjusted for the clustered nature of the data. Second, the study was not adequately powered to detect small mediation effects and was underpowered for the multiple mediation models.

Additionally, mediation analysis using Preacher and Hayes’ Multiple Mediation Regression [60] uses completers analysis for missing data, however, high retention rates at 3-month and 6-month follow-up in this study minimized the impact that this procedures has on the results. For future consideration, the study sample was relatively homogenous and future implementations of the Fit-4-Fun study should be extended in size and scope in order to represent a broader population and explore the generalizability of the study findings. Furthermore, data should be collected to assess the specific type and frequency of encouragement provided by teachers during the intervention period. Despite these limitations, the information from this study may be useful in informing future large scale Fit-4-Fun intervention implementation, and in the design of similar research projects targeting physical activity in children. Data in this area is very limited and our results will add to the growing body of research focusing on understanding physical activity behaviors in young people.

## Conclusion

Mediation analysis is an important component of physical activity research and is a useful tool in identifying the variables responsible for changes in physical activity [96]. Our study has shown that a school-based physical activity program for children (Fit-4-Fun) resulted in increased physical activity levels which were mediated by changes in teacher support for physical activity. These findings concur with research suggesting that the teacher holds the key to learning in schools [71], and suggest that researchers targeting children in the school setting should utilize the influence of the teacher in promoting positive physical activity behaviors both at school and at home. The lack of mediation effect for the other targeted variables (social support from peers and parents, enjoyment, school physical environment, self-efficacy for physical activity) should be addressed and changes in program strategies designed to modify these variables warrants further investigation.

## Abbreviations

SCT: Social Cognitive Theory; CMT: Competence Motivation Theory; HRF: Health-Related Fitness; HR: Heart rate; CRF: Cardio-respiratory fitness; MF: Muscular fitness; PA: Physical activity; PF: Physical fitness.

## Competing interests

The authors declare that they have no competing interests.

## Author contributions

Study concept and design: NE, PJM, and DRL. Acquisition of data: NE. Analysis and interpretation of data: NE. Drafting of manuscript: NE. Critical revision of the manuscript: MPJ and DRL. Statistical analysis: NE and DRL. Obtained funding: NE, DRL, PJM. All authors read and approved the final manuscript.
